# A Practical Handbook of Medical Chemistry

**Published:** 1856-05

**Authors:** 


For sale by PHINNEY & Co.

---

ART. V.—*A Practical Handbook of Medical Chemistry.* By JOHN E. BOWMAN, F. C. S., Professor of Practical Chemistry in King's College, London. Second American from the Third and Revised London Edition. With Illustrations. Philadelphia: Blanchard and Lea. 1855.

We must presume, from the fact that this is the second American edition, that our readers are not so ignorant of its existence as we confess ourselves to have been. This circumstance alone would prevent us from according to it any lengthy notice, even had we not now lying on our table, as yet unnoticed, the kindred works of Lehmahn—works whose great importance demands from us a careful examination.

But were it not for these reasons, there is much in this little duodecimo worthy of more than usual praise. It attempts one thing, and does that one thing well. Without going into any theoretical notions about the mysteries of physiological chemistry, it offers to the practitioner just what he needs to enable him to recognize the chemical characters of the various secretions, whether healthy or morbid.

It takes up, first, the subject of the urine, with its healthy and morbid conditions, giving full, yet plain directions for its qualitative analysis in its various forms of disease. This is followed by calculi and concretions, urinary, biliary and gouty. The third part is devoted to the analysis of the blood; the fourth to that of milk, mucus, pus, bone, &c.; and the fifth to the detection of poisons in organic mixtures.

